# Characterizing non-critically ill COVID-19 survivors with and without in-hospital rehabilitation

**DOI:** 10.1038/s41598-021-00246-1

**Published:** 2021-10-26

**Authors:** Benjamin Musheyev, Rebeca Janowicz, Lara Borg, Michael Matarlo, Hayle Boyle, Wei Hou, Tim Q. Duong

**Affiliations:** 1 Department of Radiology, Montefiore Medical Center and Albert Einstein College of Medicine, 111 E 210th St, Bronx, NY 10467 USA; 2 Renaissance School of Medicine at Stony Brook University, Stony Brook University, Stony Brook, NY USA; 3 Department of Physical and Occupational Therapy, Renaissance School of Medicine at Stony Brook Medicine, Stony Brook, NY USA; 4 Department of Family, Population and Preventative Medicine, Renaissance School of Medicine at Stony Brook University, Stony Brook, NY USA

**Keywords:** Health care, Medical research

## Abstract

This study investigated pre-COVID-19 admission dependency, discharge assistive equipment, discharge medical follow-up recommendation, and functional status at hospital discharge of non-critically ill COVID-19 survivors, stratified by those with (N = 155) and without (N = 162) in-hospital rehabilitation. “Mental Status”, intensive-care-unit (ICU) Mobility, and modified Barthel Index scores were assessed at hospital discharge. Relative to the non-rehabilitation patients, rehabilitation patients were older, had more comorbidities, worse pre-admission dependency, were discharged with more assistive equipment and supplemental oxygen, spent more days in the hospital, and had more hospital-acquired acute kidney injury, acute respiratory failure, and more follow-up referrals (*p* < 0.05 for all). Cardiology, vascular medicine, urology, and endocrinology were amongst the top referrals. Functional scores of many non-critically ill COVID-19 survivors were abnormal at discharge (*p* < 0.05) and were associated with pre-admission dependency (*p* < 0.05). Some functional scores were negatively correlated with age, hypertension, coronary artery disease, chronic kidney disease, psychiatric disease, anemia, and neurological disorders (*p* < 0.05). In-hospital rehabilitation providing restorative therapies and assisting discharge planning were challenging in COVID-19 circumstances. Knowledge of the functional status, discharge assistive equipment, and follow-up medical recommendations at discharge could enable appropriate and timely post-discharge care. Follow-up studies of COVID-19 survivors are warranted as many will likely have significant post-acute COVID-19 sequela.

## Introduction

Coronavirus disease 2019 (COVID-19)^1,2^ caused by the novel severe acute respiratory syndrome coronavirus 2 (SARS-CoV2) has evolved into a global pandemic. To date, SARS-CoV-2 has killed 3 million and infected 136 million worldwide (https://coronavirus.jhu.edu, Apr 12, 2021). Many SAR-CoV2 infected patients are hospitalized with debilitating illness, and some will likely require long term medical treatments and rehabilitation.

In-hospital rehabilitation that includes physical and occupational therapy has been shown to minimize hospital-acquired weakness, promote rapid functional recovery, and improve quality of life^3–5^. COVID-19 circumstances, however, have made in-hospital rehabilitation challenging for COVID-19 patients, limiting access to the types of restorative services and extent of rehabilitation. During rehabilitation, functional status can be assessed to determine where patients could be discharged, to which locations, the types of assistive equipment needed, follow-up recommendation, as well as to identify which patients may need further rehabilitative interventions. Common inpatient assessment tools of functional status include Mental status, intensive-care-unit (ICU) Mobility Scale, and Barthel Index. Mental status assesses whether a patient is alert and oriented to person, place, time, and situation^6^. The ICU Mobility Scale assesses mobility ranging from being passively rolled in bed to ambulating independently^7^. The Barthel Index evaluates the level of assistance required to complete basic activities of daily living (ADL) including feeding, toilet transfers and toileting, bathing, dressing, grooming, and stair negotiation^8^. Systematic documentation of functional status of hospitalized COVID-19 survivors at hospital discharge could help to identify patients who may need additional and timely medical or rehabilitative interventions as well as to anticipate future healthcare needs^8–17^.

In-hospital physical and occupational therapy services not only provide restorative therapies during hospitalization but also assist in discharge planning. This includes determining discharge location, durable medical equipment (DME) needs, supplemental oxygen needs, and medical follow-up referrals. Common variables included in such planning involve patient pre-COVID-19 admission dependency status, comorbidities and risk factors, functional status, and length of hospital stay amongst others. To date, data on the extent of in-hospital rehabilitation, functional status, the types of discharged assistive equipment, follow-up medical recommendations, and discharge locations of COVID-19 survivors are generally lacking.

The goal of this study was thus to characterize the pre-COVID-19 admission dependency status, discharge DME, discharge medical follow-up recommendation, discharge locations, rehabilitation status, and functional status at hospital discharge of non-critically ill COVID-19 survivors, stratified by those who received rehabilitation and those who did not. Clinical data including laboratory tests, vital signs, length of hospitalization, and hospital-acquired illnesses were also obtained for comparisons. Functional status was assessed using the Mental status, ICU Mobility Scale, and Barthel Index scores. To our knowledge, this is the first study to systematically evaluate rehabilitation status and functional status of non-critically ill COVID-19 survivors at hospital discharge and correlate them with other clinical variables.

## Results

### Clinical characteristics of rehabilitation and non-rehabilitation group

Of the non-critically ill hospitalized COVID-19 patients, 31.9% received in-hospital rehabilitation services. Electronic medical record data were extracted for 155 patients who received in-hospital rehabilitation and 162 patients (a subset) who did not receive any rehabilitation for comparison. Demographics, medical insurance status, comorbidities, symptoms, laboratory tests, and vital signs at hospital admission for the rehabilitation and non-rehabilitation groups are summarized in Table 1**.** The rehabilitation group were older and had fewer Hispanic’s compared to the non-rehabilitation group (*p* < 0.05), but there was no sex difference between groups (*p* > 0.05). The majority of the non-rehabilitation group (88%) and rehabilitation group (93%) had medical insurance. The rehabilitation group had a higher prevalence of pre-existing hypertension, coronary artery disease, immunosuppression, psychiatric disorders, arrythmia’s, thromboembolic disorders, and hypothyroidism (*p* < 0.05) compared to the non-rehabilitation group. The rehabilitation group had fewer smokers than the non-rehabilitation group. The top five most common comorbidities/risk factors (obesity, hypertension, smoking, diabetes, and coronary artery disease) were similar in both groups. There were more patients with multiple comorbidities in the rehabilitation group compared to the non-rehabilitation group. Most of the non-rehabilitation (91%) and rehabilitation group (92%) were symptomatic. Of the laboratory tests and vital signs, alanine aminotransferase, brain natriuretic peptide, hematocrit, creatinine, D-dimer, troponin, lactate dehydrogenase, lymphocytes, diastolic blood pressure, SpO_2_, and heart rate were significantly different between groups (*p* < 0.05).

**Table 1:** Demographics, comorbidities, symptoms, laboratory tests, and vitals in the rehabilitation (N = 155) and non-rehabilitation (N = 162) group at hospital admission.

	Rehabilitation	No Rehabilitation	*p* value
**Demographics**			
**Age**	76.0 (63.0, 83.0)	51.5 (38.5, 60.0)	**0.00**
**Gender**			
Male	48.4%	57.4%	0.11
Female	51.6%	42.6%	
**Ethnicity**			
Hispanic	16.8%	30.3%	**0.01**
Non-Hispanic	71.6%	56.2%	**0.00**
**Race**			
Caucasian	50.3%	51.2%	0.87
African American	3.9%	7.4%	0.17
**Insurance**	92.9%	88.3%	0.16
**Comorbidities/Risk factors**			
Hypertension	61.3%	40.7%	**0.00**
Smoking	33.6%	47.5%	**0.02**
Coronary artery disease	19.4%	10.5%	**0.03**
Immunosuppression	13.6%	4.3%	**0.00**
Psychiatric disorder	12.3%	5.6%	**0.04**
Arrythmia	11.0%	3.7%	**0.01**
Hypothyroidism	7.1%	0.6%	**0.00**
Thromboembolic disorders	7.1%	1.2%	**0.01**
Obesity	74.8%	83.7%	**0.06**
Diabetes	29.0%	21.0%	0.10
Carcinoma	14.8%	9.9%	0.18
COPD	11.6%	9.3%	0.49
Heart failure	11.6%	6.2%	0.09
Chronic kidney disease	11.0%	11.1%	0.97
Hyperlipidemia	7.7%	3.7%	0.12
Asthma	6.5%	3.7%	0.26
GI disease	5.8%	7.4%	0.57
**Symptoms**			
Fever	53.6%	67.3%	**0.01**
Myalgia	14.8%	30.3%	**0.00**
Sore throat	1.3%	5.6%	**0.04**
Shortness of breath	54.8%	60.5%	0.31
Cough	51.0%	60.5%	0.09
Fatigue	26.5%	22.8%	0.46
Nausea/vomiting	23.3%	24.1%	0.86
Diarrhea	18.7%	20.4%	0.71
Chest discomfort/pain	11.6%	16.1%	0.25
Sputum	5.8%	6.8%	0.72
Headache	4.5%	8.6%	0.14
Runny nose	3.9%	2.5%	0.48
Loss of taste	3.9%	3.7%	0.94
Loss of smell	2.6%	3.1%	0.79
Asymptomatic	8.4%	9.3%	0.78
**Laboratory tests**			
Alanine Aminotransferase, U,L	21.5 (12.0, 37.5)	34 (20.5, 66.5)	**0.01**
Brain natriuretic peptide, ng/L	529 (148, 1346)	163 (38, 541)	**0.00**
Hematocrit, %	38.5 (33.4, 41.9)	40.2 (37.0, 44.7)	**0.00**
Creatinine, mg/dL	1.29 (0.84, 35.6)	1.06 (0.72, 5.73)	**0.00**
D-Dimer, nmol/L	412 (240, 1027)	317 (201, 946)	**0.00**
Troponin, µg/L	0.01 (0.01, 0.03)	0.01 (0.01, 0.01)	**0.01**
Lactate dehydrogenase, U/L	333 (269, 401)	351 (263, 434)	**0.02**
Lymphocytes, %	12.3 (9.5, 16.5)	13 (7.3, 18.6)	**0.02**
Procalcitonin, ng/mL	0.16 (0.10, 0.24)	0.22 (0.108, 0.33)	0.08
Bicarbonate, mEq/L	24 (22, 26)	23 (21.25, 25.5)	0.26
Sodium, mEq/L	137.5 (133, 140)	136 (130, 139)	0.31
Aspartate Aminotransferase, U/L	38 (24, 55)	41 (26.5, 57.8)	0.38
White blood cells, G/L	6.63 (5.0, 9.0)	7.77 (5.6, 9.7)	0.49
C-reactive protein, mg/L	6.5 (3.4, 12.3)	6.7 (2.5, 15.4)	0.51
Ferritin, µg/L	635.4 (228, 1234)	796 (384, 1163)	0.74
**Vitals**			
Diastolic blood pressure, mmHg	72.7 (64.6, 78.6)	72.7 (68.3, 77.4)	**0.02**
SpO_2_, %	95.3 (94, 96.6)	95.9 (94.7, 97.1)	**0.02**
Heart rate, bpm	82.7 (71.5, 94.8)	92.3 (73.4, 106.9)	**0.01**
Systolic blood pressure, mmHg	130 (124, 144)	123 (115, 129)	0.08
Respiratory rate, rate/min	19.3 (17.6, 21.1)	20 (18, 24)	0.16
Temperature, °C	37.1 (36.8, 37.4)	37.3 (36.9, 37.8)	0.07
**In-hospital diagnosis**			
Acute kidney injury	10.3%	9.3%	0.75
Acute respiratory failure	20.0%	13.0%	0.09
**Functional scores [standard error of the mean]**			
Mental status	2.72 [0.05] out of 3	N.A	N.A
ICU mobility	6.87 [0.21] out of 10	N.A	N.A
Barthel Index	45.58 [2.05] out of 75	N.A	N.A

Group comparison of categorical variables in percentages used Chi-square tests. Group comparison of continuous variables in medians and interquartile ranges (IQR) used the Mann–Whitney U test. Functional scores are represented as mean [SEM]. *p* values in bold indicate statistical significance. N.A. indicates not available. COPD: chronic obstructive pulmonary disease, GI disease: Gastrointestinal disease.

### Pre-admission status

**Figure 1:**
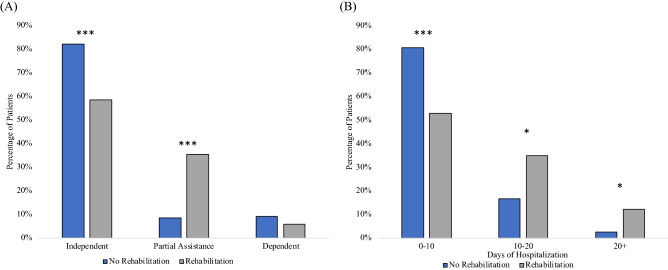
(**A**) Percentage of patients who were independent, dependent, or required partial assistance in the non-rehabilitation (N = 162) group and the rehabilitation (N = 155) group. (**B**) Percentage of patients in the non-rehabilitation (N = 162) and rehabilitation (N = 155) group as a function of lengths of stay. *** indicates significance *p* < 0.001, * indicates *p* < 0.05 (Chi square). Note that the percentages sum up to 100% for non-rehabilitation group and 100% for the rehabilitation group, separately.

In the non-rehabilitation group, 82% were independent, 9% needed partial assistance and 9% were dependent prior to COVID-19 hospitalization, whereas in the rehabilitation group, 59% were independent, 35% needed partial assistance, and 6% were dependent (Fig. 1A). There were more independent patients who did not need rehabilitation (*p* < 0.001), more partial assistance patients needed rehabilitation (*p* < 0.001), and a similar number of dependent patients needed rehabilitation (*p* > 0.05).

### Length of stay (LOS)

The non-rehabilitation group spent fewer days in the hospital compared to the rehabilitation group (5 [3, 8] vs 9 [5, 16] days, median [IQR], *p* < 0.0001). Figure 1B shows days of hospitalization for three bins. There were a higher percentage of patients who were discharged 0–10 days in the non-rehabilitation group than the rehabilitation group (81% vs 53%, *p* < 0.0001), but fewer who were discharged between 10–20 days (17% vs 35%, *p* < 0.05) and 20+ days (2% vs 12%, *p* < 0.05).

### Discharge equipment

**Figure 2:**
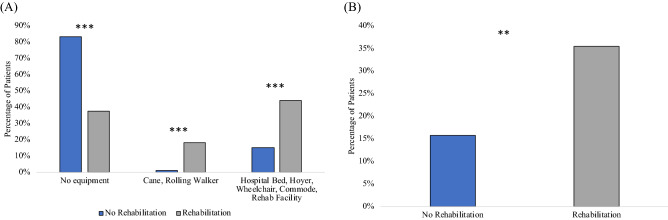
(**A**) Percentage of patients in the non-rehabilitation and rehabilitation group discharged: (i) with no equipment, (ii) with cane or rolling walker, (iii) with hospital bed, Hoyer, wheelchair, and/or commode (also referred to as durable medical equipment, DME), and to rehabilitation facility. (**B**) Patients discharged with or without supplemental oxygen equipment. *** indicates *p* < 0.001, ** indicates *p* < 0.01 (Chi Square).

Compared to the non-rehabilitation patients, fewer rehabilitation patients were discharged with no equipment (38% vs 83% *p* < 0.001), whereas more rehabilitation patients were discharged with a cane or rolling walker (18% vs 1%, *p* < 0.001), and with DME (44% vs 15%, *p* < 0.001 for all, Fig. 2A). More rehabilitation patients were discharged with oxygen equipment than the non-rehabilitation group (35% vs 16% respectively, *p* < 0.01, Fig. 2B).

### Follow-up referrals

**Figure 3:**
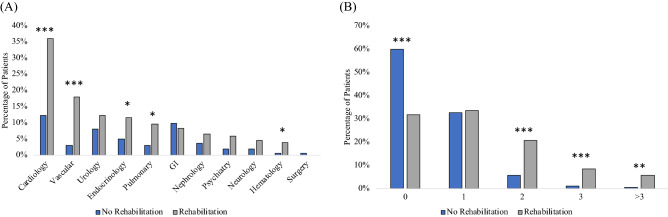
(**A**) Histogram of medical follow-up recommendations, and (**B**) percentages of patients with number of follow-up recommendations. *** indicates *p* < 0.001, ** indicates *p* < 0.01, * indicates *p* < 0.05 (Chi square).

Comparatively more referrals were observed in the rehabilitation group. Cardiology, vascular medicine, urology, and gastroenterology follow-up referrals were among the top six recommendations for rehabilitation group (Fig. 3A). Cardiology, vascular medicine, endocrinology, pulmonology, and hematology referrals were significantly different between groups (*p* < 0.05). Rehabilitation patients were more likely to have multiple referrals while the non-rehabilitation group was more likely to have no referrals (*p* < 0.001, Fig. 3B).

### Discharge locations

**Figure 4:**
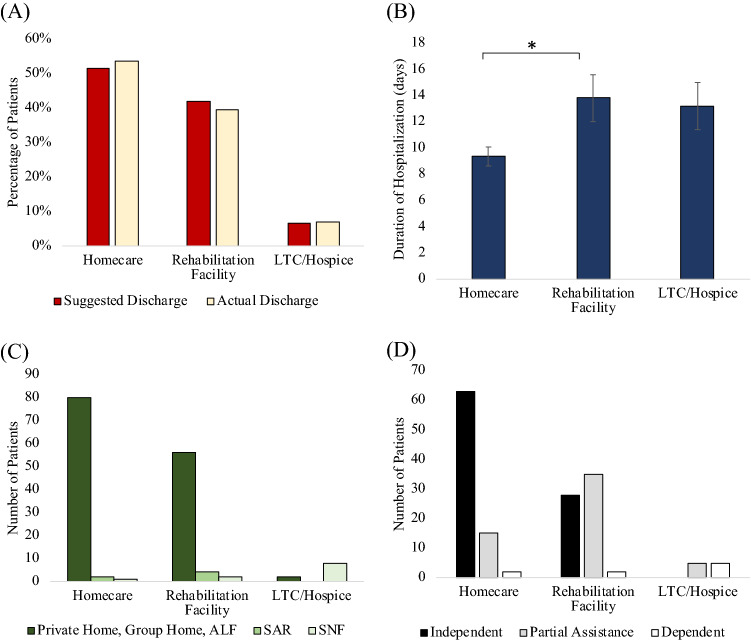
(**A**) Discharge compliance, (**B**) duration of hospitalization versus suggested discharge location, (**C**) pre-admission domicile versus actual discharge location, and (**D**) pre-admission independent status versus suggested discharge location. * indicates *p* < 0.05 (Chi Square). ALF: assisted living facility, SAR: sub-acute assisted rehab, SNF: skilled nursing facility.

Most patients adhered to their suggested discharge recommendation (*p* > 0.05, Fig. 4A). Of those who did not, 38% elected a higher standard of care than suggested and 62% elected a lower standard of care. About half (54%) of the patients were discharged to home, 39% to rehabilitation facility and < 10% to long-term care (LTC)/hospice.

Patients discharged to homecare spent significantly less time in the hosptial compared to those dicharged to rehabilitation (9.4 vs 13.8 days, *p* < 0.05, Fig. 4B). There was no significant difference in LOS between discharge location to rehabilitation and LTC/hospice (*p* > 0.05) or between between discharge location to homecare and LTC/hospice (*p* > 0.05).

For patients recommended discharge to homecare or rehabilitation, the majority (96% and 90% respectively) came from private homes, assisted living facilities (ALF), and group homes, while a few came from sub-acute rehabilitation facilities (SAR; 2% and 6%) or skilled nursing facilities (SNF; 1% and 3%) (Fig. 4C). By contrast, for patients recommended discharge to LTC/Hospice, most (80%) came from a SNF. By comparison essentially all patients in the non-rehabilitation group returned to their prior domicile.

For survivors recommended homecare, the majority were functionally independent pre COVID-19 (78.8%), some needed partial assistance (18.8%), and very few (2.5%) were dependent (Fig. 4D). For survivors recommended rehabilitation, similar number of patients were functionally independent (43%) and needing partial assistance (54%), and very few (3%) were dependent. For survivors recommended long term care or hospice, none were functionally independent, and half needed partial assistance (50%) or were dependent (50%).

### Functional scores

**Figure 5:**
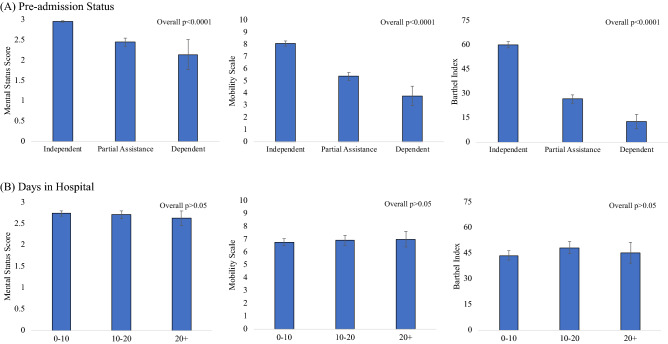
Modified Mental Status Score (range: 0–3), ICU Mobility Scale (range: 0–10), and modified Barthel Index (range: 0–75) for (**A**) pre-admission dependency status (overall *p* < 0.001, ANOVA) and (**B**) duration of hospitalization (overall *p* > 0.05, ANOVA).

Functional scores were evaluated with respect to pre-admission dependency status and LOS. Functional scores of rehabilitation patients were below normal (Mental Status Score: 2.72 out of 3, ICU Mobility Scale: 6.87 out of 10, and modified Barthel Index Score: 45.58 out of 75, all *p* < 0.0001). Higher functional scores were found in patients who were independent pre-admission, followed by those who required partial assistance and were dependent (*p* < 0.0001 for all scores, Fig. 5A). Functional scores did not depend on duration of hospitalization (*p* > 0.05, Fig. 5B).

Table 2 shows the correlation of Mental Status, ICU Mobility Scale, and modified Barthel Index scores at hospital discharge with demographics, comorbidities, laboratory tests, and vitals. Most of the Barthel scores were significantly negatively correlated with age, hypertension, coronary artery disease, chronic kidney disease, psychiatric disease, anemia, and neurological disorders (*p* < 0.05), whereas only some mobility and mental status scores were significantly correlated (*p* < 0.05). Functional scores were not significantly correlated with lung disorders, carcinoma, hyperlipidemia, gender, race, or ethnicity (*p* > 0.05).

**Table 2:** Correlation coefficients (standard errors) and *p* values of functional status scores with demographics, comorbidities, laboratory values, and vital signs.

	Mental status score	Mobility scale	Barthel Index
Hypertension	0.23 (2.88) 0.005	ns	ns
Coronary artery disease	ns	ns	0.14 (2.13) 0.035
Chronic kidney disease	− 0.20 (− 2.61) 0.01	ns	− 0.16 (− 2.35) 0.021
Psychiatric disorder	− 0.33 (− 4.33) 0	ns	− 0.15 (− 2.28) 0.024
Anemia	ns	− 0.18 (− 2.29) 0.024	− 0.18 (− 2.63) 0.01
Neurological disorders	ns	− 0.24 (− 3.22) 0.002	− 0.18 (− 2.78) 0.006
Age	− 0.27 (− 3.16) 0.002	− 0.45 (− 5.48) 0	− 0.503 (− 6.99) 0

Correlations for comorbidities that had < 6% prevalence were not analyzed as they were unreliable. ns: no significance.

## Discussion

This study characterized the non-critically ill COVID-19 survivors with respect to pre-COVID-19 admission functional status, rehabilitation status, in-hospital functional status, medical follow-up recommendation, discharge DME, discharge locations, stratified by survivors who received in-hospital rehabilitation and those who did not. The major findings are: (i) a significant number of non-critically ill COVID-19 survivors received in-hospital rehabilitation, (ii) the in-hospital rehabilitation group was older, had more comorbidities/risk factors and worse disease severity, (iii) the rehabilitation group was less independent prior to COVID-19 hospitalization and spent more days in the hospital, (iv) the rehabilitation group was discharged with more assisted equipment and more follow-up medical referrals, (v) the major follow-up references were cardiology, vascular medicine, endocrinology, pulmonology, and hematology, (vi) about half (54%) of patients who received rehabilitation were discharged to home, 39% to a rehabilitation facility and < 10% to LTC/hospice, and (vii) functional scores were associated with pre-admission dependency and some functional scores were negatively correlated with age and hypertension, coronary artery disease, chronic kidney disease, psychiatric disease, and anemia, and neurological disorders.

### Rehabilitation and non-rehabilitation patient characteristics

About a third of non-critically ill COVID-19 patients in our cohort received in-hospital rehabilitative services. The actual number of patients needing in-hospital rehabilitation were likely higher because some patients were recommended but did not receive rehabilitative services given the COVID-19 circumstances, which included but not limited to infection concern and staffing issue^18^.

It is not surprising that rehabilitation patients were older, less independent prior to COVID-19, had more comorbidities/risk factors, worse disease severity, longer LOS, discharged with more DME, and had more follow-up medical referrals. Pre-COVID-19 dependency status, age, and comorbidity are likely related and may not be independent factors accounting for rehabilitative needs. Note that the duration of hospitalization could be affected by rehabilitation facility’s policy^19^ requiring patients to be COVID-19 negative for admission and, thus, some COVID-19 patients might have remained in the hospital longer than medically necessary. Levin et al. reported significant challenges in getting patients admitted to rehabilitation facilities. They developed a system to efficiently discharge COVID-19 patients^19^ by requiring negative COVID-19 PCR testing, declining acute phase reactants (C-reactive protein, lactate dehydrogenase, ferritin, and d-dimer), supplemental oxygen requirements of < 5 L/min, and a safe post program discharge plan. Additionally, patients who had hemodialysis/peritoneal dialysis needs had limited post-acute care site choice and had a harder time being accepted in rehabilitation facilities.

The major follow-up medical referrals were cardiology, vascular medicine, endocrinology, pulmonology, and hematology, with the rehabilitation group having more follow-up referrals than non-rehabilitation group. These observations underscore physicians’ concerns of the potential post-acute COVID-19 medical issues. Although some medical follow-up referrals might have been related to pre-existing conditions, most referrals appeared to be a result of COVID-19 or worsened by COVID-19 illness. For example, vascular medicine and hematology referrals might be a concern over the hypercoagulability of COVID-19 after discharge. It has been reported that many survivors warranted extended thromboprophylaxis after hospital discharge, even for those who never had a VTE^20^. Similarly, cardiology referrals may be related to concerns of COVID-19 cardiac injury sequela, including but not limited to acute coronary injury, heart failure, myocarditis, arrhythmias, and intracardial thrombus formation^21^. Endocrinology referral is suggestive of concerns for diabetes. Steroid treatment associated with COVID-19 may be associated with worsening of diabetes and new diabetes onset^22,23^. COVID-19 circumstances could also disrupt the management of diabetes and worsen glycemic control^24^, resulting in endocrinology referral. It is surprising that pulmonology referral ranked below others given that SARS-CoV2’s primary manifestation is in the lung and survivors will likely have many long-term pulmonary sequelae^25^. It is possible that pulmonological function were largely improved by discharge and management was deferred to the primary care physician, while more acute concerns of other major organs required prompt referral.

### Discharge locations

Physical and occupational therapy services are consulted to not only provide restorative therapies in the hospital, but also to assist with discharge planning, including help to determine if a patient can return to their original domicile at time of discharge or if they would benefit from upgraded care. It is not surprising that virtually all the non-rehabilitation patients returned to their original domicile given their less severe COVID-19 course. By comparison, rehabilitation patients showed a high rate of upgraded care, a reflection of worse, clinical and functional impairment. Taken together, the needs for upgraded care and discharge location were influenced by multiple variables, including prolonged length of stay, pre-COVID-19 admission dependency status, comorbidities and risk factors, amongst others. Our findings are consistent with a previous study which reported patients who received rehabilitation services were more likely to be discharged to a rehabilitation facility while those who did not were more likely to be sent home; and longer hospitalization stay and more comorbidities are associated with discharge to a rehabilitation facility^26^.

It is surprising that 39% of patients in the rehabilitation group were discharged to a rehabilitation facility and ~ 10% to LTC/hospice. The actual percentages could be higher due to limited facilities or concerns of infection that lead patients to elect to go home^19^. While some patients came from these facilities prior to COVID-19, a significant number of patients were living independently at home. This is alarming because 90% were independent prior to COVID-19, suggesting that significant numbers of non-critically ill COVID-19 patients might not be functionally independent at least in the short term. Other explanations are possible and prospective studies are needed.

### Functional status

Patients who received rehabilitation were significantly more functionally impaired at discharge as they needed more durable medical equipment and home oxygen. This was likely due to a more severe COVID-19 course. Functional scores were not correlated with LOS. A possible explanation is that patients needed to be able to perform basic tasks and ambulate with less complicated DME before they were discharged and, thus, patients with long and short LOS could have similar functional scores^27^. Some Mental Status, some ICU Mobility Scale and most Barthel Index scores were negatively correlated with age, hypertension, coronary artery disease, chronic kidney disease, psychiatric disease, anemia, and neurological disorders. No other correlations were found between functional scores with other clinical variables. These findings suggested that functional scores were associated with pre-existing conditions or made worse by COVID-19. Follow-up studies are needed to ascertain the contribution of pre-existing conditions.

### Comparison with critically ill COVID-19 cohort

We previously reported the functional status of critically ill COVID-19 patients at discharge^28^. In that study, the majority (94.1%) were functionally independent prior to COVID-19 illness, whereas most critically ill COVID-19 patients were not functionally independent at hospital discharge (22% discharged with cane or rolling walker, 49% discharged with durable medical equipment). By comparison, 80% of the non-rehabilitation and 58% of rehabilitation group were functionally independent prior to COVID-19 illness. It is unclear why there were differences in pre-COVID-19 independency status between the critically ill and noncritically ill COVID-19 groups. It is possible that those who did not survive in the critically ill group were mostly dependent or needing partial assistance, whereas those who were functionally independent prior to COVID-19 illness were those who survived.

There were more critically ill COVID-19 patients discharged with supplemental oxygen equipment, hospital bed, Hoyer, wheelchair, or commode (durable medical equipment, DME), or discharged to rehabilitation facility, compared to both groups of noncritically ill COVID-19 patients. More critically ill COVID-19 patients were referred for medical follow-up recommendation, but the types of top medical follow-up recommendations were similar to noncritically ill COVID-19 patients.

The functional status of critically ill COVID-19 patients (Mental status = 2.7, ICU Mobility Scale = 5.75, and Barthel index = 38) showed slightly worse scores using the same tests compared to that of noncritically ill COVID-19 patients (ICU mental status = 2.72, ICU mobility scale = 6.87, and Barthel index = 48.58), as expected. Worse functional status at hospital discharge in critically ill COVID-19 patients was associated with longer invasive mechanical ventilation duration, older age, male sex, higher number of comorbidities, hypertension, diabetes, chronic obstructive pulmonary disease, and immunosuppression.

Although existing Mental status, ICU Mobility Scale, and Barthel Index scores were readily available in our electronic medical records, these scores did not assess other aspects of important function. A few studies have proposed more comprehensive tools and scoring systems to evaluate the functional status of COVID-19 patients to better predict outcomes and guide treatment after hospital discharge^29–32^. For instance, Klok et al. proposed the Post-COVID-19 Functional Status (PCFS) scale, a self-reported questionnaire that assesses patients’ ability to live independently, their functional limitations, and their degree of suffering from post-COVID-19 symptoms, pain, depression, or anxiety and categorizes patients into Grades 0–4 based on their responses^32^. The PCFS scale has the advantage of prospectively analyzing patients, categorizing them into an ordinal as opposed to interval scale, and incorporating psychological health into their analysis. However, our scores are objectively measured from electronic medical records and focus on unbiassed assessments of functional and mental status making them less susceptible to subjective error. Still, they are limited in how measures of psychological health are difficult to extract retrospectively and not all functional limitation is documented. Additional studies using more comprehensive tools to assess functional status of COVID-19 survivors are needed.

Our findings suggest that many non-critically ill hospitalized COVID-19 survivors’ ability to resume pre-COVID-19 roles and responsibilities were negatively impacted at least in the short, and possibly, long term^33^. Other studies have found that impaired functional status extended well beyond hospital discharge as patients were reporting persistent symptoms that impact their functional abilities six months after disease onset^29–31,34–36^. Early rehabilitation has been shown to improve physical and cognitive recovery in COVID-19 patients^33^. There need to be adequate rehabilitation programs to accelerate recovery for COVID-19 survivors^29,33^.

### Limitations

The strength of this study is that it is the first study to systematically characterize in-hospital rehabilitation, discharged recommendation, discharged equipment, and functional status of non-critically ill COVID-19 survivors. This study however has several limitations. The sample is small and, thus, these findings and conclusions might not be generalizable to the population at large. Multi-site and larger cohort studies are needed. The small sample sizes could impact some findings (such as discharge locations where patients were subdivided into three groups) more than others. Correlation of scores with clinical variables is also more susceptible to small sample size. As with all retrospective study, there could be unintended patient selection bias and there could be other residual confounders that were not accounted for in our analysis. In-hospital functional assessment was constrained by COVID-19 circumstances. Patients were usually confined to their hospital rooms to reduce cross-infection, and thus ambulation distance for functional assessment was limited. Many hospitals, including ours, needed to increase bed capacity during the peak of COVID-19, resulting in shortage of some rehabilitation equipment and reduction in room space, making rehabilitation challenging. The COVID-19 circumstance also confined patients to beds or rooms, limiting mobility and resulting in worse functional scores at discharge which likely negatively impact long-term physical recovery. Due to these constraints, certain components of Barthel Index could not be scored and thus the modified Barthel Index used in this study only had a range of 0–75. Our study only evaluated COVID-19 patients in-hospital settings. Prospective studies are warranted to follow up COVID-19 patients after hospital discharge using more comprehensive scoring systems as many patients will likely have significant post-acute COVID-19 sequela^35^.

### Conclusions

A significant number of non-critically ill COVID-19 survivors received in-hospital rehabilitation and many survivors showed impaired functional status that was below prior level at hospital discharge, suggesting many non-critically ill COVID-19 survivors will likely need additional medical care after hospital discharge. Knowledge of COVID-19 patient functional status, discharge assistive equipment, and medical recommendations at hospital discharge is important because it enables appropriate follow-up care in a timely manner. Our study contributes to better understanding the functional status of noncritically ill COVID-19 survivors, which can help facilitate more appropriate follow-up care in a timely manner. Further follow-up studies of COVID-19 survivors are warranted as many will likely have significant post-acute COVID-19 sequela.

## Methods

This retrospective study was approved by the Stony Brook University Institutional Review Board with an exemption for informed consent. This study followed the Strengthening of Reporting of Observational Studies in Epidemiology (STROBE) reporting guidelines for cross-sectional studies (http://www.equator-network.org/reporting-guidelines/strobe/). All methods were performed in accordance with the relevant guidelines and regulations. Data were obtained from the emergency room at Stony Brook University Hospital between March 27, 2020 and August 11, 2020. A subset of this database has been used previously to address different research questions^37–49^. COVID-19 was confirmed based on a real-time polymerase chain reaction test for severe acute respiratory syndrome coronavirus 2 (SARS-CoV-2) on a nasopharyngeal swab specimen. Exclusions were COVID-19 positive patients who were: (i) not hospitalized, (ii) had incidental COVID-19 findings but were admitted for other major medical indications (i.e., trauma), (iii) still in the hospital at the time of the study, (iv) less than 18 years old, and (v) admitted to the ICU. Electronic medical record data were extracted for 155 patients in the rehabilitation group and 162 patients (a subset) in the non-rehabilitation group.

### Demographics, comorbidities and laboratory variables

Demographics (age, gender, ethnicity, and race), comorbidities, pre-admission dependency status, medical insurance status, laboratories tests and vital signs were tabulated for the rehabilitation and non-rehabilitation group. Chronic comorbidities and risk factors included smoking, hypertension, diabetes, asthma, chronic obstructive pulmonary disease, coronary artery disease, heart failure, cancer, and chronic kidney disease, amongst others. Symptoms included fever, shortness of breath, cough, myalgia, nausea/vomiting, fatigue, diarrhea, chest discomfort/pain, headache, amongst others. Chronic comorbidities/risk factors and symptoms were collected from patient electronic medical records that were either self-reported at hospital admission or obtained from prior diagnoses listed in the electronic medical records. Laboratory tests at hospital admission included C-reactive protein, D-dimer, ferritin, lactate dehydrogenase, lymphocytes, procalcitonin, alanine aminotransferase, aspartate aminotransferase, and troponin, amongst others. Vital signs included heart rate, respiratory rate, pulse oxygen saturation [SpO_2_], systolic blood pressure and temperature at hospital admission. In addition, the prevalence of in-hospital acquired acute kidney injury (AKI), acute respiratory failure, and acute respiratory distress were also tabulated.

### Pre-admission dependency

Pre-admission dependency status was obtained from the care management comprehensive assessment notes in the medical record that tabulated patients as independent, partial assistance or dependent status for the rehabilitation and non-rehabilitation group. In cases where pre-admission dependency status was not obviously stated, the overall narrative of the care management notes was considered to stratify patients. Independent patients were those who did not require any physical assistance for mobility or to perform activities of daily living (ADL) prior to hospital admission. Patients in the partial assistance group required some form of physical assistance for mobility or ADL. Dependent patients were those who could not function independently and completely relied on assistance for physical activity and ADL. Length of stay (LOS) was also tabulated for the rehabilitation and non-rehabilitation group.

### Discharge equipment and notes

The following discharge data were obtained: (i) discharge equipment (1: none, 2: cane/walker, and 3: hospital bed, Hoyer, wheelchair, or commode (durable medical equipment, DME), or discharge to rehabilitation facility), (ii) discharge with or without supplemental oxygen equipment, (iii) discharge follow-up recommendations (i.e., cardiology, vascular medicine, pulmonology, endocrinology, neurology, urology, hematology, surgery, gastroenterology, nephrology, psychiatry, ophthalmology, orthopedics/rheumatology, and wound care). Follow-up recommendations of infectious disease and primary care physicians were common to essentially all patients and were not plotted.

### Compliance, length of stay, pre-admission dependency status, and pre-admission domicile with respect to discharge location

Discharge data were obtained for suggested and actual discharge location (1: homecare, 2: rehabilitation facility, 3: long-term care (LTC), or hospice). Compliance, LOS, pre-admission domicile status, and pre-admission dependency status with respect to discharge location were analyzed for the rehabilitation and non-rehabilitation group. LOS was binned 0–10, 10–20 and ≥ 21 days; such bins were chosen to average out daily fluctuations. Binning daily or so did not alter trends and conclusions. For pre-admission domicile status, patients were grouped into (i) those from private homes, assisted living facilities (ALF), and group homes, (ii) those from a sub-acute rehabilitation facility (SAR), and (iii) those from a skilled nursing facility (SNF).

### Functional scores

Functional status was assessed retrospectively using inpatient functional scores which included the Mental status, ICU Mobility Scale, and Barthel Index^28^. The modified Mental Status score (range: 0–3) assesses alertness, orientation and ability to follow commands. One point is given if the patient is alert but not oriented, two points are given if the patient is alert and oriented to at least two domains (self, location, time, or situation), and an additional point is given if the patient is able to follow commands. The ICU Mobility Scale (range: 0–10) is an 11-item categorical scale that measures the highest level of functional mobility of patients. Although ICU mobility scale is used in the ICU setting, it is appropriate for this study because COVID-19 patients were constrained to hospital rooms due to COVID-19 circumstances. The Barthel Index (original range: 0–100) is an ordinal scale used to measure performance in ADL, consisting of ten variables describing ADL and functional mobility, with a higher number reflecting greater ability to function independently. Due to the isolation precautions for patients with COVID-19, therapy sessions were confined to the patient’s room which limited potential ambulation distances. The “mobility on level surfaces” subscale of The Barthel Index (0–15 points) could not be scored as the minimum distance must be 50 yards, greater than what is feasible within the confines of a patient room. Additionally, the “stairs” subscale of the Barthel Index (0–10 points) could not be consistently scored because it was only completed if stairs were a barrier to discharge. Thus, these subscales were eliminated, and the modified Barthel Index used for this study had a range of 0–75. Higher scores indicate higher functioning for all three scores.

Chart reviews to extract scores included reviews done by occupational therapy notes, physical therapy notes, nursing flowsheets, care management notes, medicine team notes, and speech-language pathology notes if needed. If specific notes and/or information was not available from the actual date of hospital discharge, the closest note prior to the actual date was used. During these chart reviews, COVID-19 diagnosis was confirmed as the final primary diagnosis on the patient chart. Each patient’s medical chart was rated by two independent raters^28^.

### In-hospital rehabilitation

The extent of in-hospital rehabilitation varied depending on each patient’s activity tolerance. Physical and occupational therapy services were consulted by the primary medical team when the patient was deemed to need rehabilitative services or if there was a question of whether a patient was capable of discharging home based on their clinical presentation. Patients were evaluated by physical and occupational therapists to determine a discharge disposition, and subsequently placed on a treatment program to be seen 2–3 times per week. The duration of treatment sessions was dependent on a patient’s activity tolerance. Content included bed mobility (supine to sit, sit to supine), sitting tolerance (both static and dynamic), upper extremity exercises across a spectrum from active assisted range of motion for lower level patients through active resistive exercise with therapy bands and weights, breathing exercises targeting increased cardiopulmonary endurance, trunk strengthening, sit to stand practice, transfer training (bed to chair, several steps at bedside, short ambulatory transfers), basic ADLs (grooming, toileting, donning/doffing clothing), and leg exercises both in seated and standing.

### Statistical analysis

Statistical analysis was performed using SPSS v26 (IBM, Armonk, NY) and SAS v9.4 (SAS Institute, Cary, NC). Chi-square was used to compare categorical demographics, comorbidities, and symptoms at presentation between the rehabilitation versus non-rehabilitation group. Paired t-tests were used to compare functional scores to baseline. Age, laboratory tests, vital signs and in-hospital diagnosis were compared using Mann–Whitney U test between the rehabilitation versus non-rehabilitation group. Suggested discharge locations were compared with actual discharge locations of the rehabilitation group using McNemar’s test. Rehabilitation and non-rehabilitation group differences in pre-admission independency status, LOS and discharge location, discharge equipment, and medical follow-up recommendation used Chi-square analysis.

Functional scores of patients who received rehabilitation were compared across pre-admission status and the number of days of hospitalization using an ANOVA. For all analyses, a p < 0.05 was considered statistically significant.

## Data Availability

The datasets generated during and/or analyzed during the current study are available from the corresponding author on reasonable request.
